# A Small Ligand That Selectively Binds to the G-quadruplex at the Human Vascular Endothelial Growth Factor Internal Ribosomal Entry Site and Represses the Translation

**DOI:** 10.3389/fchem.2021.781198

**Published:** 2021-11-09

**Authors:** Xiao-Xia Hu, Sheng-Quan Wang, Shi-Quan Gan, Lei Liu, Ming-Qing Zhong, Meng-Hao Jia, Fei Jiang, Yan Xu, Chao-Da Xiao, Xiang-Chun Shen

**Affiliations:** ^1^ State Key Laboratory of Functions and Applications of Medicinal Plants, Guizhou Medical University, Guiyang, China; ^2^ Department of Physiology, College of Basic Medical Sciences, Guizhou Medical University, Guiyang, China; ^3^ Division of Chemistry, Department of Medical Sciences, Faculty of Medicine, University of Miyazaki, Miyazaki, Japan; ^4^ The Key Laboratory of Optimal Utilization of Natural Medicine Resources, Guizhou Medical University, Guiyang, China

**Keywords:** G-quadruplex, VEGF, selective ligand, translation, RNA

## Abstract

G-quadruplexes are believed to have important biological functions, so many small molecules have been screened or developed for targeting G-quadruplexes. However, it is still a major challenge to find molecules that recognize specific G-quadruplexes. Here, by using a combination of surface plasmon resonance, electrospray ionization mass spectrometry, circular dichroism, Western blot, luciferase assay, and reverse transcriptase stop assay, we observed a small molecule, namely, oxymatrine (OMT) that could selectively bind to the RNA G-quadruplex in 5′-untranslated regions (UTRs) of human vascular endothelial growth factor (hVEGF), but could not bind to other G-quadruplexes. OMT could selectively repress the translation of VEGF in cervical cancer cells. Furthermore, it could recognize VEGF RNA G-quadruplexes in special conformations. The results indicate that OMT may serve as a potentially special tool for studying the VEGF RNA G-quadruplex in cells and as a valuable scaffold for the design of ligands that recognize different G-quadruplexes.

## Introduction

G-quadruplexes are four-stranded structures found in DNA/RNA sequences that are rich in guanine residues. Compared with double-helical DNAs, G-rich RNAs are relatively unconstrained and easily form G-quadruplexes (Bugaut and Balasubramanian, 2012). Both antibody and fluorescence probes have been developed to detect the existence of RNA G-quadruplex in cells (Biffi et al., 2014; Liu et al., 2016; Xu et al., 2016). X-ray and NMR analyses have indicated that the RNA sequences can form G-quadruplexes in different conformations (Xu et al., 2008; Collie et al., 2010; Xu et al., 2010; Xiao et al., 2017a; Xiao et al., 2017b; Xiao et al., 2018). Accumulating evidence has revealed that the RNA G-quadruplex has important biofunctions, such as regulating telomere length, forming telomeric heterochromatin, and protecting telomeres (Rippe and Luke, 2015; Montero et al., 2016; Koch, 2017; Abraham Punnoose et al., 2018). Moreover, a bioinformatics analysis suggested that as many as 3000 5′-UTRs of mRNAs may contain sequences that are possible to form G-quadruplexes (Kumari et al., 2007). Experimental data proved that RNA G-quadruplexes within such regions exerted important regulatory roles for gene expression (Bugaut and Balasubramanian, 2012). A well-known example is the RNA G-quadruplex in the 5′-UTR of human vascular endothelial growth factor (hVEGF) mRNA. The 17-nucleotide (nt) sequence r (GGA​GGA​GGG​GGA​GGA​GG) in the internal ribosomal entry site (IRES) region was identified adopting the G-quadruplex structure. The G-quadruplex is called the VEGF RNA G-quadruplex in the following sections. The VEGF RNA G-quadruplex can recruit the 40S ribosomal subunit directly, which is essential for the initiation of cap-independent translation (Morris et al., 2010; Bhattacharyya et al., 2015). Ligands stabilizing the VEGF RNA G-quadruplex can repress the expression of VEGF (Cammas et al., 2015). Other RNA G-quadruplexes, such as those within mRNAs of BCL-2/NRAS, also play important regulatory roles in the expression of proteins. Owing to its significant biological role, the G-quadruplex is considered an ideal target for ligand development. Recently, a number of molecules targeting the G-quadruplex have been reported, such as BRACO19, pyridostatin, Phen-DC3, L2H2-6OTD, and L1H1-7OTD (Burger et al., 2005; De Cian et al., 2007; Rodriguez et al., 2008; Tera et al., 2008; Tera et al., 2009; Katsuda et al., 2016; Neidle, 2017; Herdy et al., 2018; Asamitsu et al., 2019b). Some of these molecules have shown anticancer effects and have been evaluated in clinical trials (Drygin et al., 2009; Xu et al., 2017). However, owing to the high structural similarity among G-quadruplexes and the complexity of cells, most of the ligands mentioned above lack selectivity for a particular G-quadruplex. As G-quadruplexes widely prevail, the low selectivity of ligands may cause unexpected side effects and cell cytotoxicity, which is usually the reason for the halting of the drug development process (Asamitsu et al., 2019a; Asamitsu et al., 2019b). Molecule selectivity binding toward individual G-quadruplexes is worth exploring as it can exert specific biological functions of the relevant G-quadruplex with minimal off-target effects.

**FIGURE 1:**
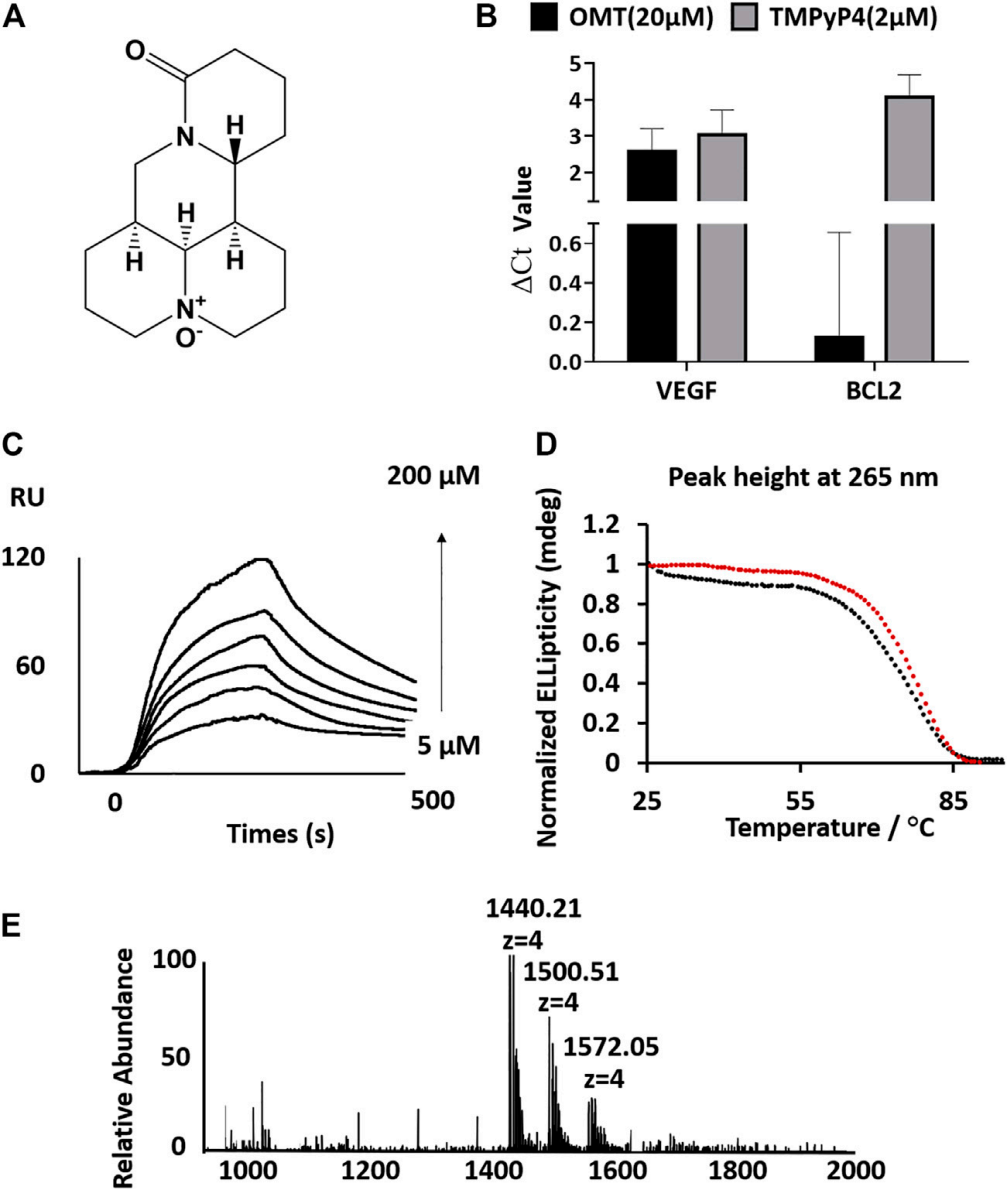
**(A)** Structure of OMT. **(B)** Analysis of the full-length cDNA production by qPCR. ΔCt values were determined by subtracting the Ct value of qPCR with the compound from the Ct value of qPCR without the compound. A high ΔCt value indicates strong inhibition of reverse transcription. OMT reduced the production of full-length cDNA from a VEGF RNA G-quadruplex containing the RNA template. **(C)** Sensorgrams (resonance units vs time) of SPR analysis for the binding of OMT on the VEGF RNA G-quadruplex. The interaction was recorded at different concentrations of OMT (final concentration: 5, 10, 20, 50, 100, and 200 μm). **(D)** CD melting curves of the VEGF RNA G-quadruplex with (red circles, Tm value: 76.3°C, standard error of fitting: 0.2564) or without (black circles, Tm value: 72.7°C, standard error of fitting: 0.4382) OMT (3:1 OMT/G-quadruplex molar ratio). **(E)** ESI-MS spectra of the VEGF RNA G-quadruplex with OMT (3:1 OMT/G-quadruplex molar ratio).

Accumulating evidence suggests that oxymatrine (OMT) (Figure 1A), a natural product isolated from *Radix sophorae flavescentis*, can efficiently repress the expression of VEGF and has an effective anticancerous effect (Chen et al., 2013; Halim et al., 2019; Jung et al., 2019). Here, we found that OMT could selectively bind to the VEGF RNA G-quadruplex. The binding between OMT and the VEGF RNA G-quadruplex *in vitro* was confirmed and characterized through surface plasmon resonance (SPR), electrospray ionization mass spectrometry (ESI-MS), and circular dichroism (CD) melting studies. The results showed that OMT was a highly specific binder for the VEGF RNA G-quadruplex. No affinity of OMT was found with other G-quadruplexes or with DNA double strands. Furthermore, the results of luciferase assays confirmed the binding of OMT with the VEGF RNA G-quadruplex in cells. Western blot experiments showed that OMT selectively repressed the expression of the VEGF protein. SPR and luciferase experiments also showed that OMT can recognize the VEGF RNA G-quadruplexes in different conformations. Altogether, OMT showed recognizability for the VEGF RNA G-quadruplex. The results may provide a valuable scaffold for further developing the ligand binding–intended G-quadruplex with high specificity, which may serve as the specific inhibitor for VEGF.

## Materials and Methods

### Reverse Transcriptase–Based Stop Assay

The sequences for reverse transcription were ordered and purchased from GenePharma^TM^, China. Detailed sequence information is described in Supplementary Table S1. The templates were purified using an NAP-5 column first. Then, the purified template RNAs (100 nM) were heated to 95°C for 3 min and gradually the temperature (1°C/min) was lowered to ambient temperature to allow G-quadruplex formation. The reaction solution was prepared with compounds (final concentration: 10 µM) mixed with the templates and incubated for 10 min at 25°C. RT primers [5′-CATGGTTTCGGAGGCCCGACCGGG-3′] for the VEGF RNA G-quadruplex template and [5′-CATCCTTCCCAGAGGAAAAGC-3′] for the BCL-2 RNA G-quadruplex template were added, respectively, to the corresponding reaction solution, and ReverTra Ace reverse transcriptase (EasyScript^TM^), MgCl2, and dNTPs were then introduced into the reaction mixture. Then, the reaction mixtures with the reacting solutions were placed into an incubator (37°C, 15 min). The reaction product (1 µL) was used as a template for the next quantitative PCR (qPCR) analysis. The primers for qPCR were designed as a forward primer [5′-GCTAGCTCGGGCCGGGAGGA-3′] with a reverse primer [5′-CATGGTTTCGGAGGCCCGACCGGG-3′] for the VEGF RNA G-quadruplex, and the forward primer [5′-TAATACGACTCACTATAGGG-3′] with the reverse primer [5′-CATCCTTCCCAGAGGAAAAGC-3′] for the BCL-2 RNA G-quadruplex template. Then, qPCR analysis was performed on a CFX Connect^TM^ detection system (BIO-RAD) with SYBR® Green. All ΔCt values were calculated by subtraction of the Ct value of qPCR with the compound from the Ct value without the compound.

For the RTase reaction-based stop assay, a reaction mixture of template RNA (0.3 µM), OMT (varying concentration) and the fluorescently labeled primer (5′-FAM-TAATACGACTCACTATAGGG-3′, 0.1 µM) was kept at 80°C for 3 minutes, followed by ReverTra Ace reverse transcriptase (TOYOBO), MgCl2, and dNTP introduction into the reacting solution at room temperature, and left for 30 min. The product was subsequently analyzed using 1.2% agarose gel.

### Melting Curve Measurement

CD spectra/melting curves for G-quadruplexes were evaluated using the AVIV model 430 CD spectrophotometer. The samples (HPLC-purified and desalted oligonucleotides) were purchased from Takara™ and GenePharma™. The oligonucleotides were maintained at 95°C for 5 minutes, followed by a gradual return to ambient temperature. The melting curves were obtained by monitoring at the 265/295-nm CD band. The CD spectra were recorded from 220 to 320 nm. The Tm value of each sample was calculated by the following method: ellipticity was plotted with a function of temperature and fitted in GraphPad Prism 7 software using a nonlinear sigmoidal dose-response model with a variable slope. The solutions for CD and UV spectra were prepared as 0.45-ml samples (10 μM) in the presence of KCl/NaCl (100 mM) with Tris buffer (10 mM, pH 7.4). Melting curves of duplex DNA analysis with or without OMT was performed on a Shimadzu model UV2600 UV–Vis spectrophotometer with a temperature controller. The melting curves were acquisitioned by monitoring at 265-nm band by scanning continuously from 40 to 75°C.

### Surface Plasmon Resonance (NICOYA) Analysis

All binding studies based on the SPR phenomenon were performed on a two-channel open SPR instrument (Nicoyalife^TM^, Canada). All 5′-biotin–labeled and annealed nucleotides (1000 RU) were purchased from Takara^TM^ and introduced into the running buffer (10 mM HEPES pH 7.4, 100 mM KCl). Nucleotides were immobilized on the biotin sensor chip surface using a biotin–streptavidin sensor chips kit obtained from Nicoyalife^TM^, Canada. Flow cell 1 served as the control surface, where no target oligonucleotides were captured. Diluted OMT solutions (final concentration: 5, 10, 20, 50, 100, and 200 μM) were set up with the running buffer. The samples were then injected at a flow rate of 25 μL/ min during the association phase at 25°C. Data were fitted with TraceDrawer software.

### Mass Spectrometry

The electrospray ionization (ESI) mass spectra were measured, under the condition modified from the previous report, using an Exactive Orbitrap® mass spectrometer (Thermo Scientific™, United States) in the negative ion mode. (Marchand and Gabelica, 2014). Dataset collection employed Xcalibur® (Thermo Scientific™). A final strand concentration of 10 μM of the RNA oligomer was mixed with 30 μM OMT in the presence of 50% methanol, for preparing the G-quadruplex/OMT complex solution. Trimethylammonium acetate (TMAA) (final concentration: 100 mM) was mixed with the G-quadruplex/OMT complex solution prior to injection. The ESI spray/capillary voltages were 2.75 kV and -20 V, respectively, with a capillary temperature of 150°C. The ion accumulation time was 100 ms. All samples were infused within the ESI source (20 μL/minute). During titration assays, the VEGF RNA G-quadruplex (10 μM) was mixed with OMT at different concentrations. The tube lens/skimmer voltages were maintained at –180 and –10 V, respectively. As a facilitating measure for de-solving, HCD cell voltage was maintained at 10 eV and cell pressure was maintained at 2.5 × 10^−5^ mbar (Marchand et al., 2016).

### Plasmid Construction

To generate the bicistronic constructs containing VEGF IRES, we constructed a pcDNA3.1(+)-Rluc-IRES-Fluc plasmid. Briefly, the two luciferase genes, namely, Renilla luciferase (RLuc) and firefly luciferase (FLuc), were controlled by the cytomegalovirus promoter (CMV) and separated only by the VEGF IRES sequence (nts 746–1038) (Cammas et al., 2015). The restriction sites used were BamHI and EcoRI, which were added into the two sides of the sequence separately. We first synthesized the sequences encompassing the Renilla luciferase, VEGF IRES fragment, and firefly luciferase (Rluc-VEGF IRES -Fluc) by the PCR assembly approach. Synthetic DNA assemblies were performed using the GoldenBraid 2.0 DNA assembly framework (Sarrion-Perdigones et al., 2019). The full-length Rluc-VEGF IRES -Fluc sequence was divided into two parts (fragment A and B). Oligonucleotides (in Supplementary Table S2,) were ordered and purchased from GenePharma P1 to P34 were used for the PCR assembly of fragment A. The oligonucleotides can overlap each other at least by 20 nt and were (each oligonucleotide concentration: 0.5 µM) dissolved in a reaction mixture solution in the presence of 10×Pfu buffer (containing 2 mmol/L Mg^2+^), dNTP (concentration: 200 μmol/L), and 5 U Pfu DNA polymerase. The PCR assembly was conducted as follows: denaturation at 95°C for 3 min followed by 30 cycles at 94°C for 30 s, 55°C for 30 s, and 72°C for 30 s and termination by incubation at 72°C for 5 min. The product from the PCR reaction assembly was further amplified using primers P1 and P34 in a 50-µL reaction solution containing 0.5 µL of the PCR assembly mixture, 200 μmol/L of each dNTP solution, 0.5 μmol/L of each primer, 2.5 U Pfu DNA polymerase, and 10 × Pfu Buffer (containing 2 mmol/L Mg^2+^). PCR was conducted as follows: denaturation at 95°C for 3 min followed by 30 cycles at 94°C for 30 s, 55°C for 30 s, and 72°C for 30 s and termination by incubation at 72°C for 5 min. Fragment B was synthesized under the same conditions with the oligonucleotides P33 to P68 and amplified with the primers P33 and P68. Fragments A and B were purified using agarose gel. The vector pcDNA3.1(+) Invitrogen™ was digested with the enzymes BamHI and EcoRI (Fermentas). Fragments A and B were finally cloned into pcDNA3.1(+) using the ClonExpress® Entry One Step Cloning Kit. The resulting plasmid was named as pcDNA3.1(+)-Rluc-IRES-Fluc. Mutant plasmids were conducted using mutant sequences (Figure 5C) in PCR assembly procedures. All plasmids were verified by sequencing performed by Takara.

### Cell Culture and Luciferase Assay

On Day 0, HeLa cultures were incubated on 6-well plates at 1.0 × 10^6^ cells per well. On day 1, the cells were exposed to transfection vectors using the Lipofectamine™ 2000 Transfection Reagent (Invitrogen^TM^,United States), according to the manufacturer’s protocol. On day 2, such cells were co-incubated with OMT. Following 24 h of incubation, the cell cultures in each well were subjected to lysis, and activities of firefly/Renilla luciferases were gathered using the Dual-Luciferase^®^ Reporter Assay System (Promega^TM^, United States). The enzyme activities were normalized (firefly luciferase normalized against Renilla luciferase) accordingly.

### Western Blot Analysis of VEGF, NRAS, and BCL-2 Expression

HeLa cultures were exposed to OMT for 2 days. Consequently, cell lysis employed Solarbio LIFE SCIENCES™ R0010® buffer (Estonia). The lysis solution was centrifuged at 12,000 × g/min (20 min/4°C), and the supernatant was extracted. The samples were segregated over 10% SDS-PAGE gel and consequently placed onto a PVDF membrane. Subsequently, membrane immunoblotting was performed, using specific antibodies for VEGF, BCL-2, and NRAS (Santa Cruz Biotechnologies™, United States; 1:1,000 dilution); and specific antibodies for GAPDH (Abcam^TM^ Cambridge, United States; 1:10,000 dilution). The immunodetecting step was performed using the secondary antibody/ECL. Proteomic expression profiles were quantitatively analyzed through Odyssey (LI-COR™) or enhanced chemiluminescence (Pierce™).

### QRT-PCR Measurements for Evaluating the mRNA Level of VEGF in HeLa Cells

HeLa cultures were exposed to OMT for 2 days. RNA extraction employed the TRIzol® kit (Transgen Biotech™). The reverse transcription step employed EasyScript® one-step gDNA removal and cDNA synthesis super mix. qRT-PCR runs were conducted over the CFX Connect^TM^ detection system (BIO-RAD^TM^, United States) using the following primers: F (5′-TGC​ATT​GGA​GCC​TTG​CCT​TG-3′); R (5′-CGG​CTC​ACC​GCC​TCG​GCT​TG-3′) for VEGF and F (5′-GCA​CCG​TCA​AGG​CTG​AGA​AC-3′); and R (5′-TGG​TGA​AGA​CGC​CAG​TGG​A-3′) for GAPDH, the latter serving as the reference/normalization gene.

### Cytotoxicity Assays

HeLa cultures were exposed to varying OMT doses for 2 days, with OMT cytotoxicity being consequently evaluated by the use of the CellTiter-Glo1 Luminescent Cell Viability Assay^®^ (Promega™, Madison, WI, United States). The analyses employed GraphPad Prism® Software (Prism™, United States).

## Results and Discussion

### The OMT Molecule Particularly Binds to the VEGF RNA G-Quadruplex

To test whether OMT can bind to G-quadruplexes, we used the reverse transcriptase (RTase) reaction-based method modified from a previous study to compare the binding of OMT with different G-quadruplexes (Supplementary Figure S1, Supplementary Table S1) (Katsuda et al., 2016). We mixed OMT with an RNA template containing the RNA G-quadruplex and compared the inhibition efficiency of RTase reactions, in which the well-known G-quadruplex selective binder TMPyP4 was used as a positive control. Molecules binding to the RNA G-quadruplex can efficiently inhibit the elongation of the reverse transcriptase (RTase) reaction, so as to reduce the production of the full-length complementary DNA (cDNA) strand (Hagihara et al., 2010). Therefore, we used the two well-studied VEGF RNA G-quadruplex and BCL-2 RNA G-quadruplex as the targeted RNA G-quadruplexes (Kumari et al., 2007; Herdy et al., 2018). By monitoring the full-length cDNA production by qPCR, we found that TMPyP4 could inhibit both the RTase reactions of the VEGF RNA and BCL-2 RNA G-quadruplexes (∆Ct value higher than 2, which theoretically indicates that the inhibition rate of the RTase reaction is higher than 75%). In contrast, OMT only inhibited the RTase reaction of the VEGF RNA G-quadruplex with a ∆Ct value of 2.5, which has no effect on the BCL-2 RNA G-quadruplex. This indicates that OMT may have selectivity toward the VEGF RNA G-quadruplex (Figure 1B) and encouraged us to further investigate OMT. To verify whether the interruption of the RTase reaction was dependent on the binding of OMT to the G-quadruplex, we also performed an *in vitro* RTase reaction-based stop assay. As shown in Supplementary Figure S2, with the addition of OMT, the full-length production was reduced while the short-length production increased, which indicates that OMT inhibited the elongation of the reverse transcriptase (RTase) reaction via binding with the G-quadruplex.

Next, we investigated the binding affinity of OMT with the VEGF RNA G-quadruplex using SPR under previously described experimental conditions (Rosu et al., 2008). The results showed that the binding of OMT with the VEGF RNA G-quadruplex yielded the K_D_ value estimated as 3.75 × 10^−5^ M, whereas no SPR signal was observed for BCL-2 nor NRAS RNA G-quadruplexes (Figure 1C and Supplementary Figure S3, Supplementary Table S3).

The melting temperature (Tm) of the G-quadruplex measured by CD in the presence or absence of OMT showed that OMT increased the Tm of the VEGF RNA G-quadruplex by 3.6°C (Figure 1D, Supplementary Figure S4). In contrast, results showed that OMT had no detectable stabilization effect or binding to other G-quadruplexes (Supplementary Figure S5). Moreover, the DNA double strand also showed no binding with OMT (Supplementary Figure S6). Both the results indicate that OMT can selectively bind and stabilize the VEGF RNA G-quadruplex.

**FIGURE 2:**
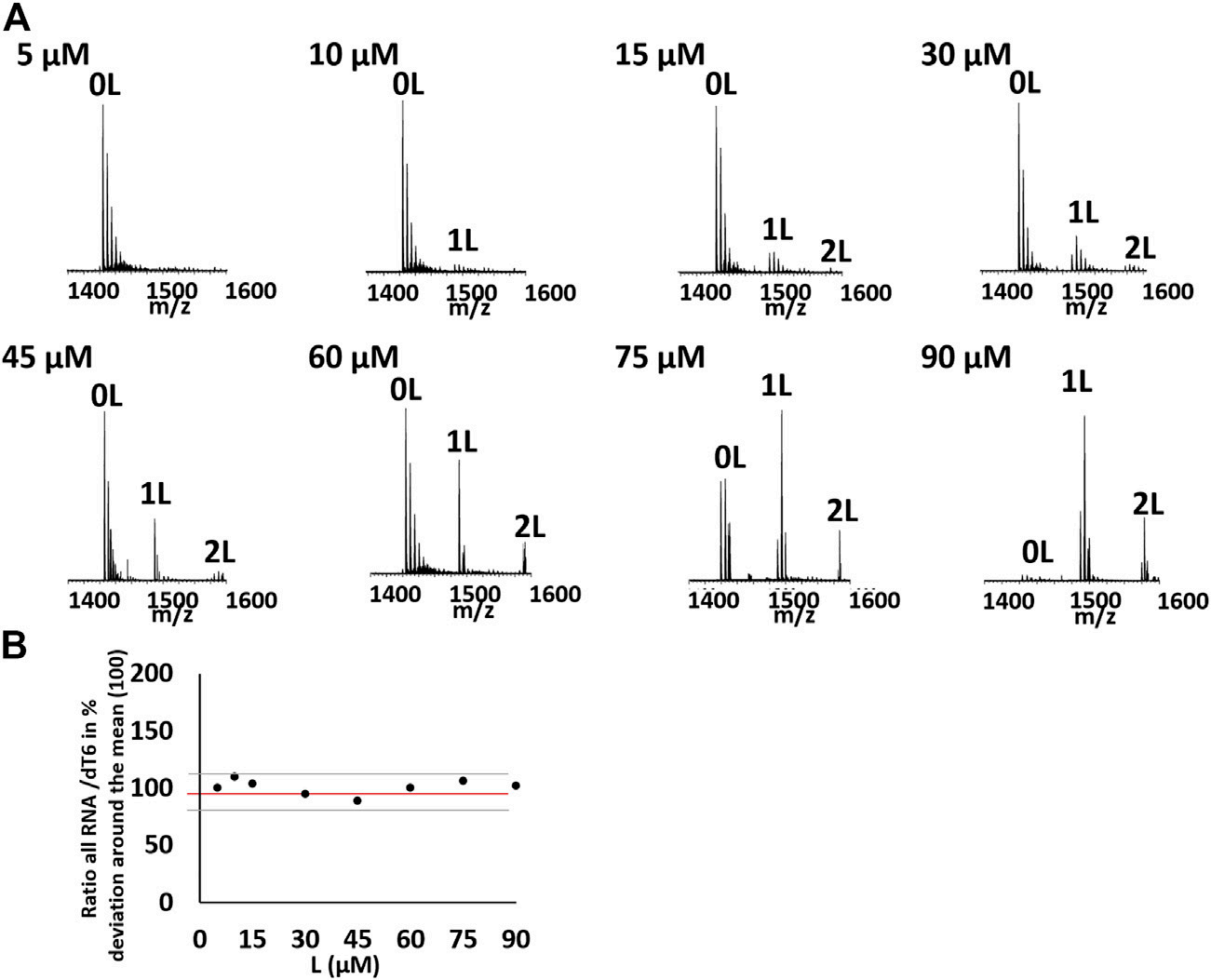
**(A)** Titration of 10 µM of the hVEGF RNA quadruplex with OMT at different concentration (5, 10, 15, 30, 45, 60, 75, and 90 μM) in 100 mM of TMAA. Zooms on the 4-charge state. Peaks corresponding to the G-quadruplex/ligand (1:1/1:2) were labeled with 1 and 2 L, respectively. **(B)** Evolution of the ratio between the total RNA signal of the 4-charge state and the internal standard dT6 in the function of the ligand concentration in percentage of deviation around the mean for the hVEGF RNA quadruplex with OMT at different concentrations. Black represents the experimental points, red represents the mean, and gray represents the standard deviation. There is no systematic deviation from the mean value on titration.

The ESI-MS is a useful method for the quantitative study of noncovalent complexes (Rosu et al., 2008; Marchand et al., 2016). Therefore, we used ESI-MS to further characterize the stoichiometry of OMT binding with the VEGF RNA G-quadruplex. Owing to soft ionization, the ESI technique can determine both the mass of the G-quadruplex and the mass of the G-quadruplex molecule complex with minimal fragmentation, which makes it very easy to measure the stoichiometry of the complex (Jaumot and Gargallo, 2012). As shown in Figure 1E, both the peaks (m/z 1500.51 and m/z 1572.05) corresponding to the VEGF RNA G-quadruplex/OMT complex were directly observed, in which the ratio of VEGF RNA G-quadruplex/OMT were 1:1 and 1:2, respectively. This result further confirmed the binding between OMT and the VEGF RNA G-quadruplex. We also performed ESI-MS titrations of the VEGF RNA G-quadruplex with OMT at different concentrations. We used dT6 as the internal standard and TMPyP4 as the positive control (Figure 2 and Supplementary Figure S7). The results showed that the peaks corresponding to the ligand/G-quadruplex were gradually increased with the addition of OMT. However, for the mutant sequence which was not able to form the G-quadruplex structure, no binding peaks could be observed (Supplementary Figure S8). This result once again confirmed the binding between OMT and the VEGF RNA G-quadruplex. The abovementioned results suggest that OMT can specifically interact with the VEGF RNA G-quadruplex.

### OMT Binds the VEGF RNA G-Quadruplex and Represses the VEGF Expression in Cancer Cells

**FIGURE 3:**
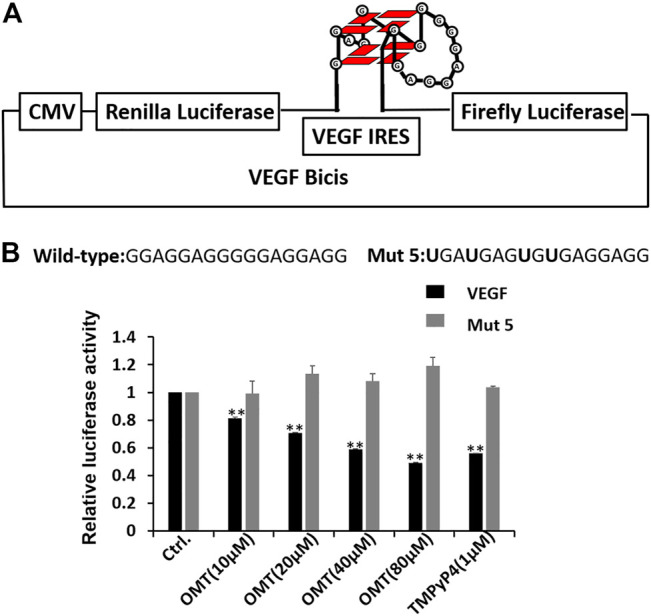
**(A)** Dual-luciferase bicistronic construct designs. The VEGF RNA G-quadruplex was inserted between the Renilla and firefly luciferases. To detect the effect of OMT on G-quadruplexes, the sequence forming the VEGF RNA G-quadruplex was also changed to the sequence with G to U mutations. **(B)** VEGF IRES activity was assessed through HeLa cell culture transfections using bicistronic transcripts carrying the IRES-A wild-type or mut 5 sequence, followed by treatment with OMT at differing concentrations. Sequence information of the wild-type and mut 5 was observed. The mutated nucleotides are displayed in bold characters. TMpyP4, a well-known G-quadruplex binder, was used as the positive control. At least three independent experiments were performed, and error bars represent standard deviation. Data were expressed as the mean ± SEM (*n* = 3). **p < 0.01 versus the control group.

Encouraged by the results of experiments *in vitro*, we next tested whether OMT can bind to the G-quadruplex and regulate the protein expression in cells. First, we prepared a dual-luciferase construct, in which the entire VEGF IRES was cloned between Renilla and firefly luciferases (Figure 3A). Although the two genes were encoded in a single mRNA, firefly translation was initiated from VEGF IRES, while renilla translation was under the control of a CMV promoter. This allowed us to quantify the effect of OMT on IRES activity in the cell. As shown in Figure 3B, OMT suppressed the translation activity of wild-type IRES, while it had no effect on the mutant model (mut 5) in which 4 G to U mutations were used to eliminate any possibility for the sequence to form intramolecular G-quadruplex. Taken together, the results suggest that OMT could bind to the G-quadruplex structure in HeLa cells.

**FIGURE 4:**
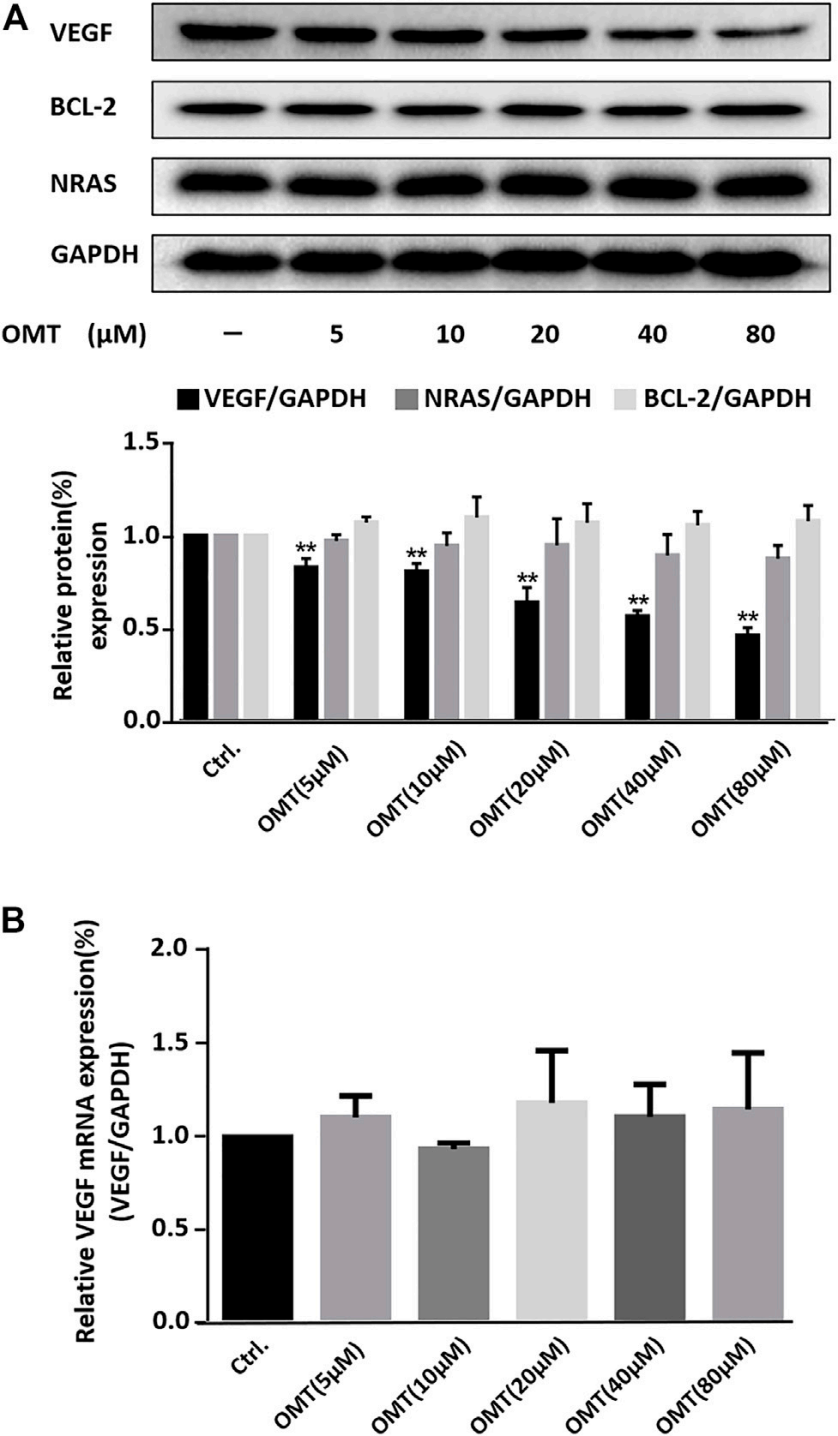
**(A)** Western blot analysis of VEGF, BCL-2, and NRAS protein expression in HeLa cells treated with OMT. GAPDH served as the loading control. Down-side histograms showed the percentage of repression of VEGF expression by OMT. Data were expressed as the mean ± SEM (*n* = 3). **p < 0.01 versus the control group. **(B)** qRT-PCR assessment for VEGF expression within HeLa cells treated with differing OMT doses was conducted and the values were consequently plotted.

Next, we performed western blot analysis to determine the effect of OMT on the expression levels of several proteins. As shown in Figure 4A, OMT caused the suppression of the expression of the VEGF protein in a dose-dependent manner. However, it had no significant effect on the expressions of the BCL-2 or on the NRAS protein, for which G-quadruplexes were also present at their 5′-UTRs (Bugaut and Balasubramanian, 2012; Varshney et al., 2020). This result again confirms the selectivity of OMT. We also examined the effect of OMT on the amount of VEGF mRNA in treated cells by quantitative reverse transcription PCR (qRT-PCR) and found no detectable effect (Figure 4B). As the G-quadruplex also existed in the promoter region of the VEGF gene, the result of qRT-PCR indicates that OMT has no effect toward the VEGF DNA G-quadruplex or DNA double strands, which is consistent with the CD and UV melting temperature results. The results of cytotoxicity assays showed that the cytotoxicity of OMT was very low (CC_50_: 10.3 mM) compared with TMPyP4 (16.35 µM) (Supplementary Figure S9) (Cheng and Cao, 2017). Such results indicate that the repression of VEGF by OMT was not due to its cytotoxicity and that OMT has high selectivity for the VEGF RNA G-quadruplex, which may be the reason for its low cellular toxicity (Neidle, 2017). According to the CD spectral result (Supplementary Figure S4), the slight shift in the positive peak indicates that the binding of OMT may distort the structure of the VEGF RNA G-quadruplex. It may arrest the access of ribosomes to the mRNA at the translation initiation step and result in the inhibition of the translation.

### OMT can Recognize the VEGF RNA G-Quadruplex in Different Conformations

**FIGURE 5:**
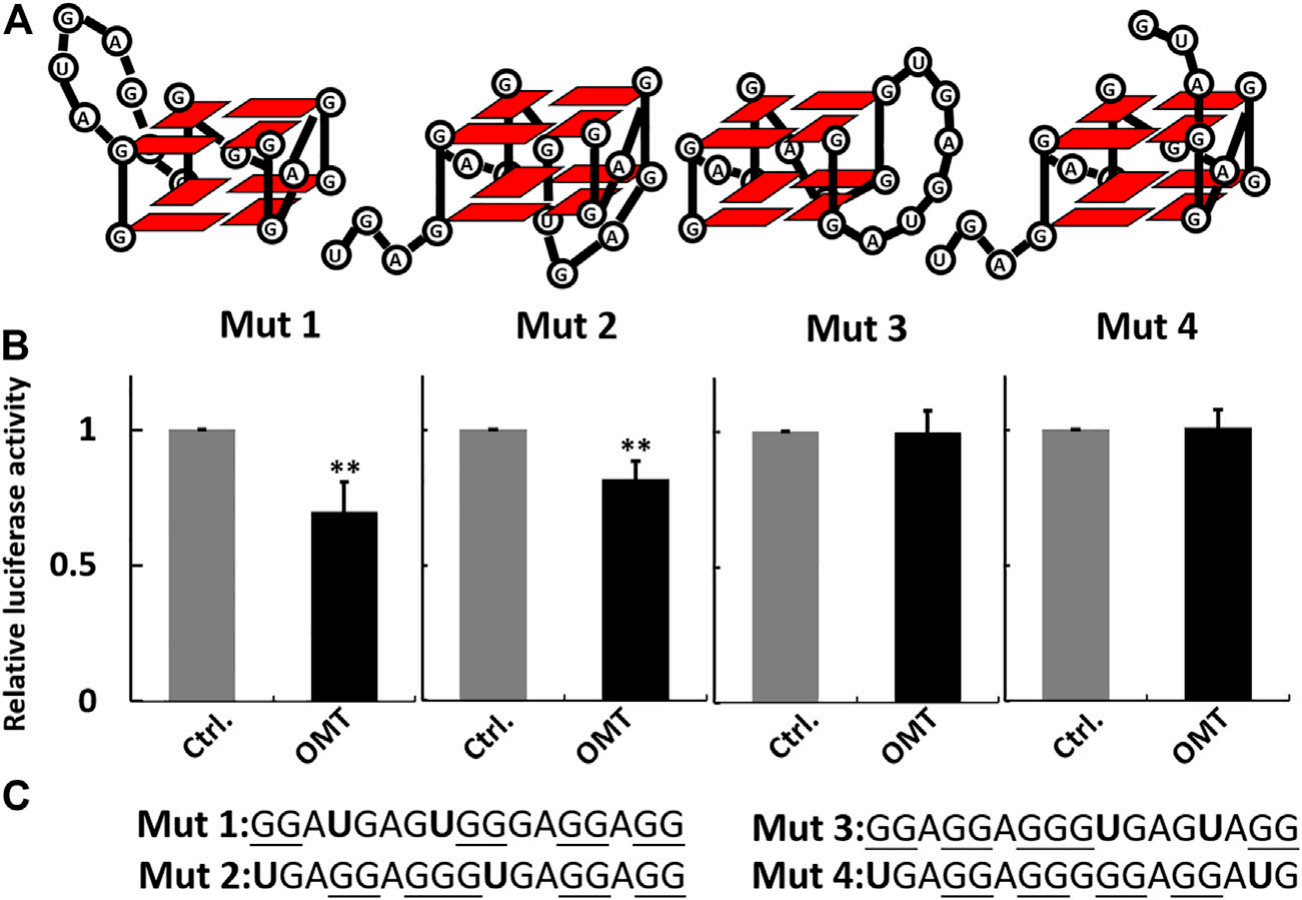
**(A)** Schematic representation of the G-quadruplexes in different conformations formed by mutant sequences. **(B)** Histogram showing percentage activity of the mutant constructs treated with OMT (40 µM). **(C)** Sequence information of the mutant sequence used in the luciferase assay. The guanosines that potentially participate in G-quadruplex formation are underlined. The mutated nucleotides are shown in bold characters. Data were expressed as the mean ± SEM (*n* = 3). **p < 0.01 versus the control group.

The binding selectivity of OMT toward the VEGF RNA G-quadruplex leads to the possibility that OMT can recognize the different conformations of RNA G-quadruplexes. As previously reported, the VEGF RNA G-quadruplex is a kind of “tunable” G-quadruplex. With different combinations of G-tracts in the sequence segment, the 17-nt sequence can form G-quadruplexes in different conformations. Several mutations of G to U can force the sequence to adopt G-quadruplexes in specific conformations, and such G-quadruplexes exert different effects on VEGF IRES activity. Based on that, we generated a series of mutated sequences as previously reported (Figure 5A) (Morris et al., 2010). Then, we incorporated them, respectively, into the dual-luciferase constructs and measured the effect of OMT on the activity of mutated IRES. The results showed that OMT could influence the IRES activity of mut 1 and mut 2, while it had no significant effect on mut 3 or mut 4. This result suggests that OMT recognizes G-quadruplexes formed by mut 1 and mut 2 (Figures 5B,C).

**FIGURE 6:**
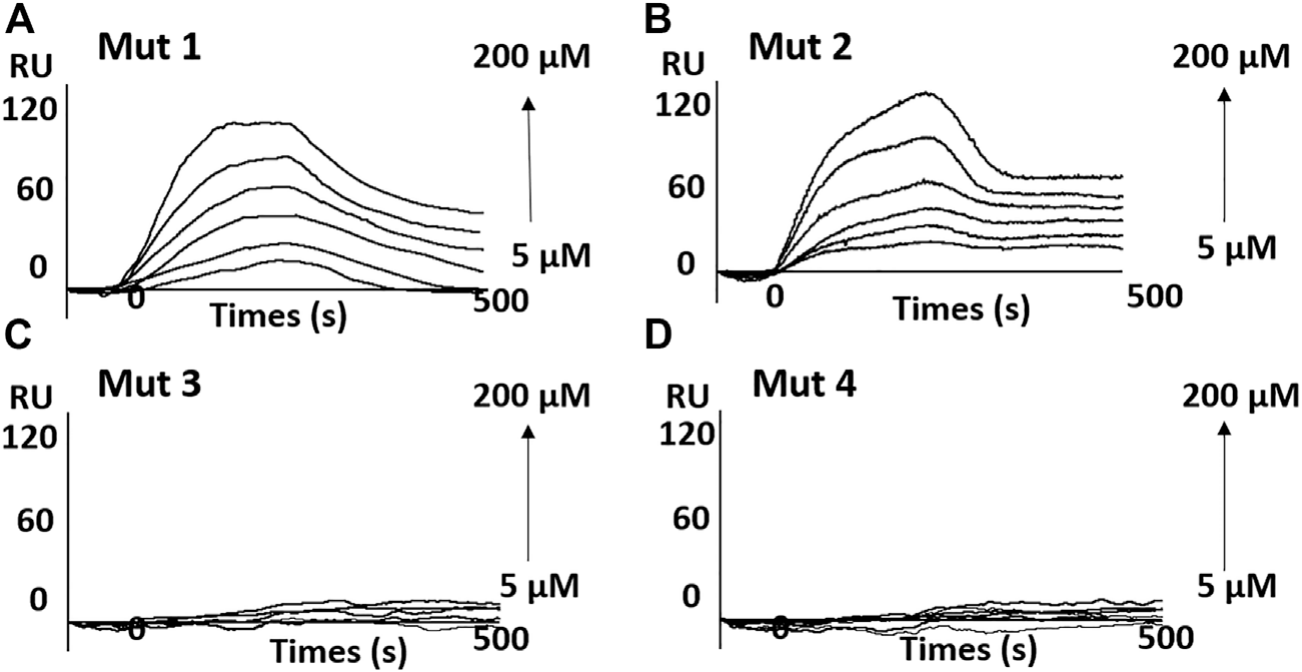
Sensorgrams (resonance units versus time) for the binding of OMT on the G-quadruplex adopted by mutant sequences. **(A)** mut 1; **(B)** mut 2; **(C)** mut 3; and **(D)** mut 4.

SPR experiments also presented similar results. As shown in Figure 6 and Supplementary Figure S10, the results indicate an obvious binding of OMT to mut 1 and mut 2, whereas it showed no signal for mut 3 or mut 4. The results further suggest that OMT may recognize the structural difference between RNA G-quadruplexes.

The OMT structure is distinct from the previously characterized G-quadruplex ligands. It is nonplanar and non-aromatic. In some reported literatures, nonplanar and non-aromatic molecules are also described as potent G-quadruplex ligands, such as the steroid derivatives (Brassart et al., 2007) and alkaloids (Li et al., 2009). The ketocarbonyl group and aminylium ion in OMT may form hydrogen bonds or electrostatic interactions with nucleotides, which may provide the affinity basis for the G-quadruplex. While the asymmetric and rigid structure of OMT may specifically accommodate into the specific pocket, the long special loop of the VEGF RNA G-quadruplex may potentially form such pockets which may contribute to the selectivity of OMT toward the VEGF RNA G-quadruplex. However, as per our knowledge, there are no NMR or crystal structures of the VEGF RNA G-quadruplex that have been reported. Further structural analysis would benefit the determination of their precise mode of interaction and clarify the molecular basis of the selectivity of binding.

## Conclusion

G-quadruplexes are considered a potential biomedical target. Finding molecules that selectively bind to the intended G-quadruplex and exhibit special activity is required. Here, we discovered OMT as the first selective binder for the VEGF RNA G-quadruplex. Both results of CD, SPR, and luciferase assays and ESI-MS showed the ability of OMT in binding to the G-quadruplex structure, and results also showed that OMT could recognize the VEGF RNA G-quadruplex in different conformations. OMT showed very low cellular toxicity, which makes it potentially promising for the drug development process. OMT affected the expression of the VEGF protein with the mRNA level of VEGF unchanged, which indicates that the translation is controlled by interaction of the VEGF RNA G-quadruplex with OMT. Thus, OMT may serve as a special tool for understanding the VEGF RNA G-quadruplex in the cell and help in the design of the ligands that recognize different G-quadruplexes.

## Data Availability

The original contributions presented in the study are included in the article/Supplementary Material; further inquiries can be directed to the corresponding authors.
