# Supplementary material for: A Small Ligand That Selectively Binds to the G-quadruplex at the Human Vascular Endothelial Growth Factor Internal Ribosomal Entry Site and Represses the Translation
Source: Front Chem. 2021 Nov 9;9:781198. doi: 10.3389/fchem.2021.781198 (PMC8630693; doi:10.3389/fchem.2021.781198)
Supplement: Supplementary file 1 [file DataSheet1.DOCX]

Supplementary Material

A small ligand that selectively binds to the G-quadruplex at the human vascular endothelial growth factor internal ribosomal entry site and represses the translation

Xiao-Xia Hu^1,4†^, Sheng-Quan Wang^1†^, Shi-Quan Gan^1^, Lei Liu^1^, Ming-Qing Zhong^1^, Meng-Hao Jia^1^, Fei Jiang^1^, Yan Xu^3^, Chao-Da Xiao^1,2*^, Xiang-Chun Shen^1,2*^

^1^ State Key Laboratory of Functions and Applications of Medicinal Plants, Guizhou Medical University, University Town, Guian New District, Guiyang, 550025, China

^2^ The Key Laboratory of Optimal Utilization of Natural Medicine Resources, Guizhou Medical University, University Town, Guian New District, Guiyang, 550025, China

^3^ Division of Chemistry, Department of Medical Sciences, Faculty of Medicine, University of Miyazaki, 5200 Kihara, Kiyotake, Miyazaki, 889-1692, Japan

^4^ Department of Physiology, College of Basic Medical Sciences, Guizhou Medical University, University Town, Guian New District, Guiyang, 550025, China

^†^These authors have contributed equally to this work and share first authorship

*** Correspondence:**Corresponding Author: Chao-Da Xiao and Xiang-Chun Shen
Email addresses: xcd@gmc.edu.cn; sxc@gmc.edu.cn


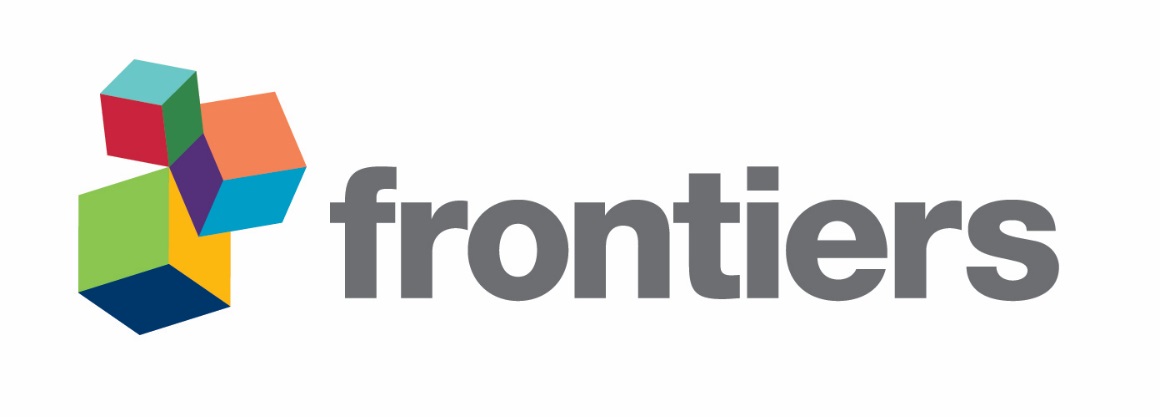


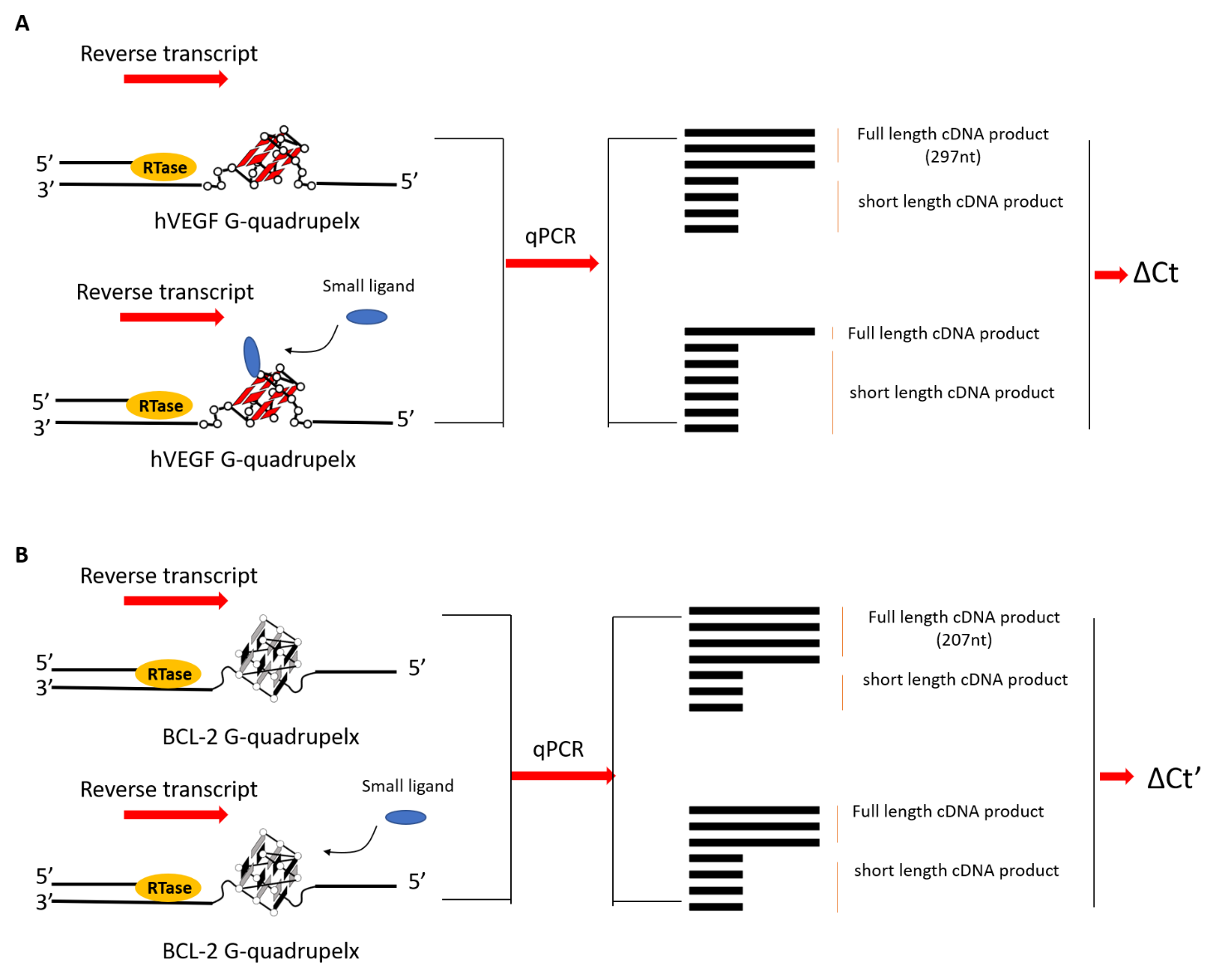


**Supplementary Figure 1.** Screening of RNA G-quadruplex stabilizers based on RTase-reaction. RNA G-quadruplex stabilizer reinforced the blocking of reverse transcriptase elongation resulting in decreased production of full-length cDNA. (a) VEGF RNA G-quadruplex was inserted into the reverse transcript template and treated with or without small ligand. Subtracted the Ct value of qPCR with compound from the Ct value of qPCR without the compound resulting in ΔCt. (b) BCL-2 RNA G-quadruplex was used as the target RNA G-quadruplex also. ΔCt′ value was compared with the ΔCt to determine the possible selectivity of OMT.


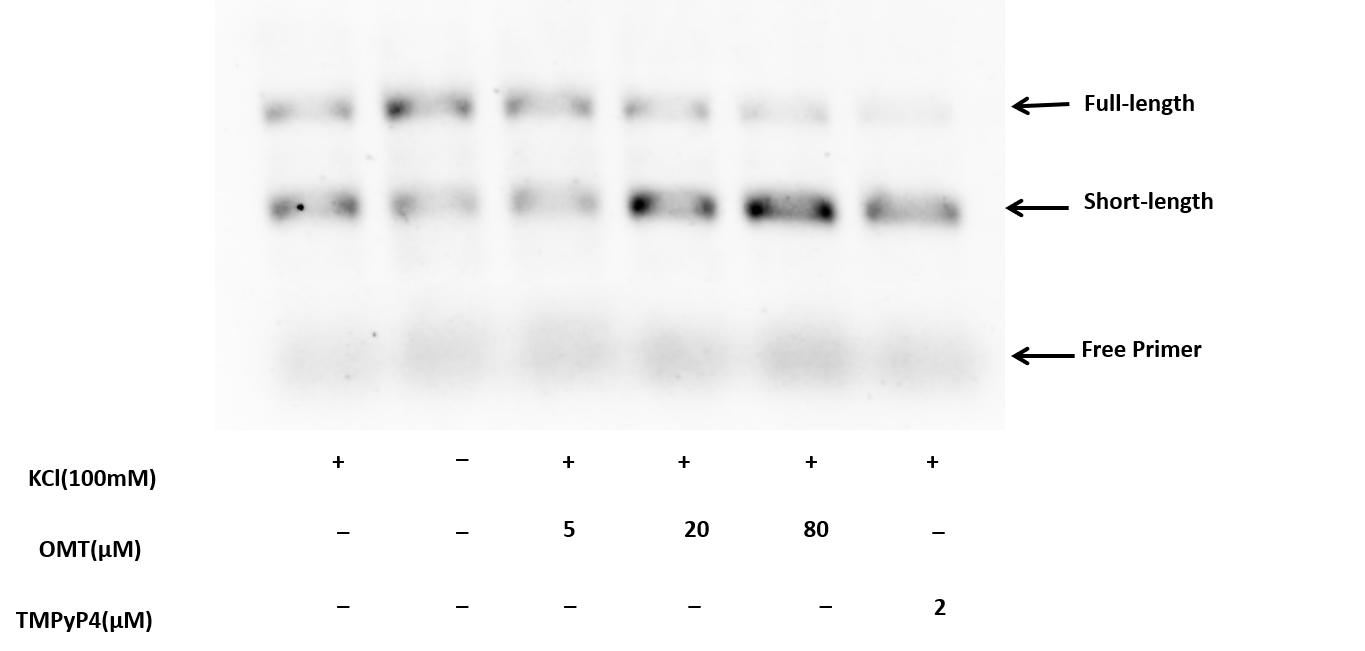


**Supplementary Figure 2.** RTase reaction-based stop assay of VEGF RNA G-quadruplex containing template treated with OMT. With addition of OMT, the short length cDNA product was increasing with the full-length product decreased. The template without stabilization of KCl was used as the negative control. TMPyP4 was used as the positive control


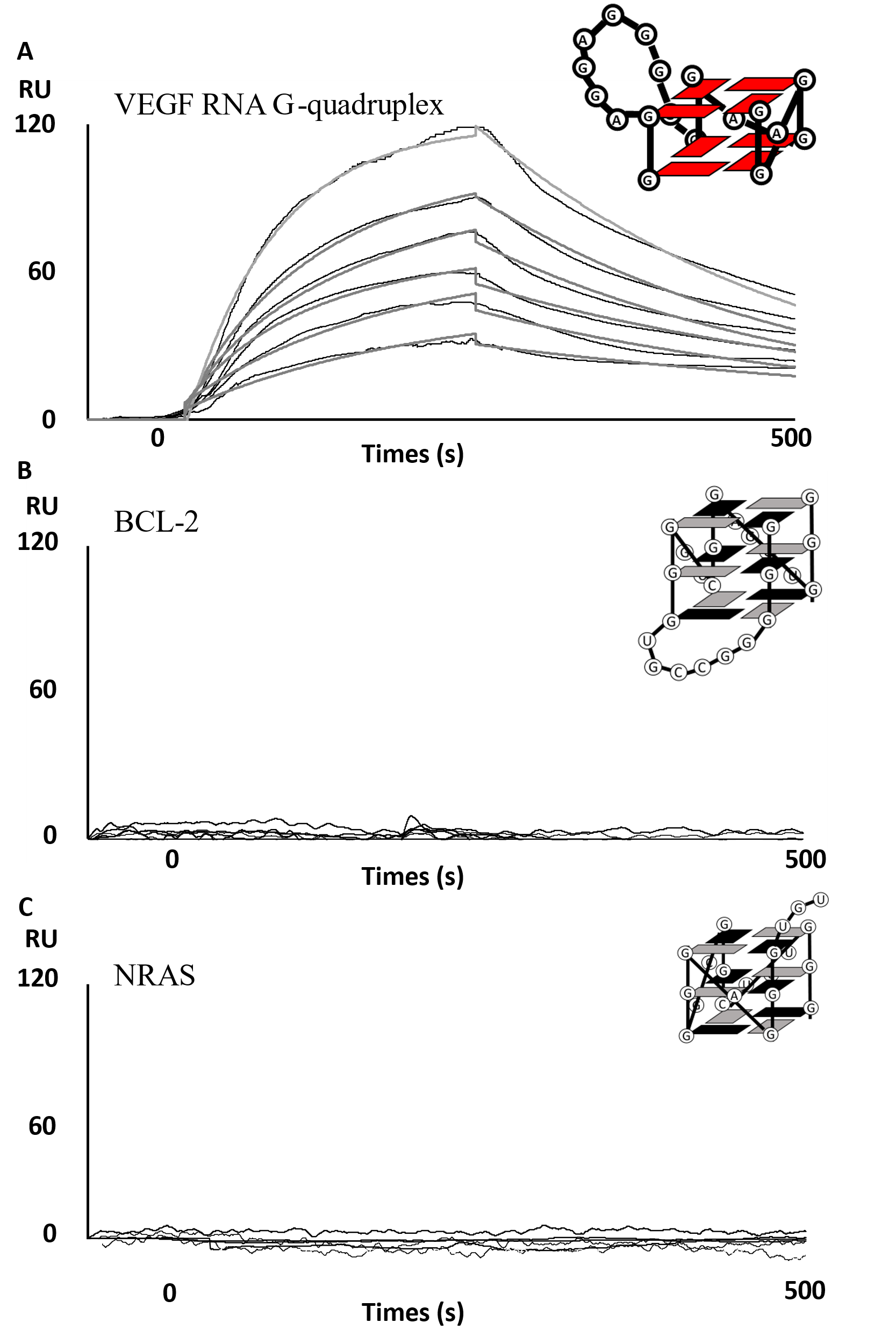


**Supplementary Figure 3.** SPR sensorgrams for binding of OMT with VEGF RNA G-quadruplex. Experimental data was in black and fitted curves in gray. (A) The data was fitted with TraceDrawer software by one-to-one model. (B) The data of OMT binding with BCL-2 RNA G-quadruplex. (C) The data of OMT binding NRAS RNA G-quadruplex.


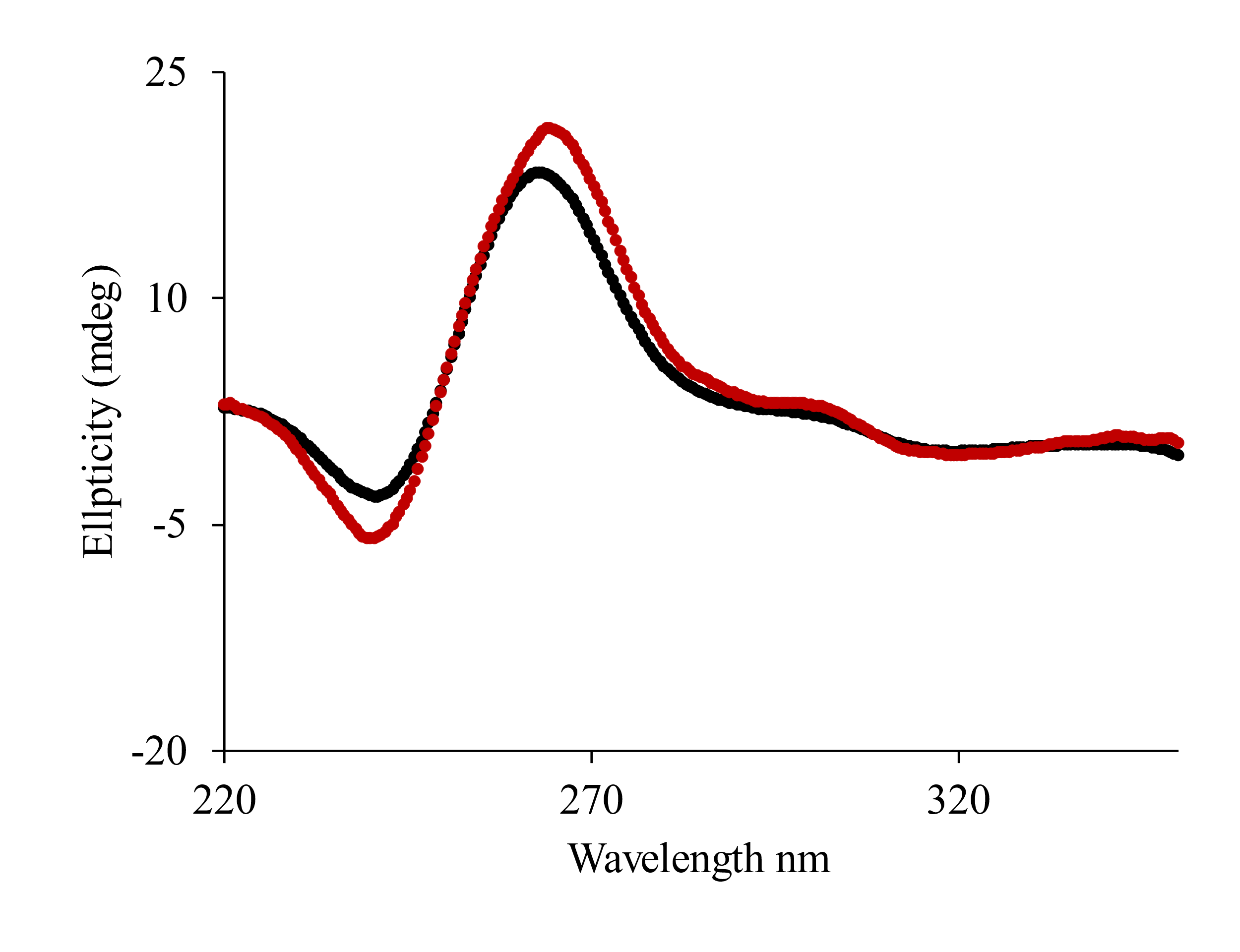
**Supplementary Figure 4.** The CD spectra of VEGF RNA G-quadruplex with (red circles) or without (black circles) OMT in 10 mM Tris-HCl buffer (pH 7.4) containing 100 mM KCl at 25 °C.

**
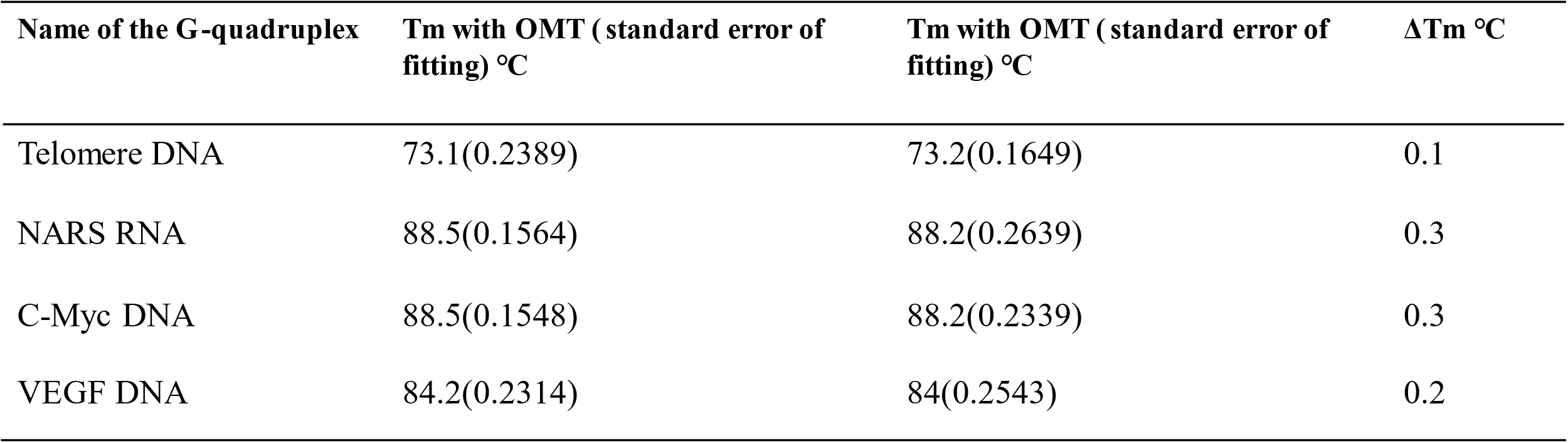

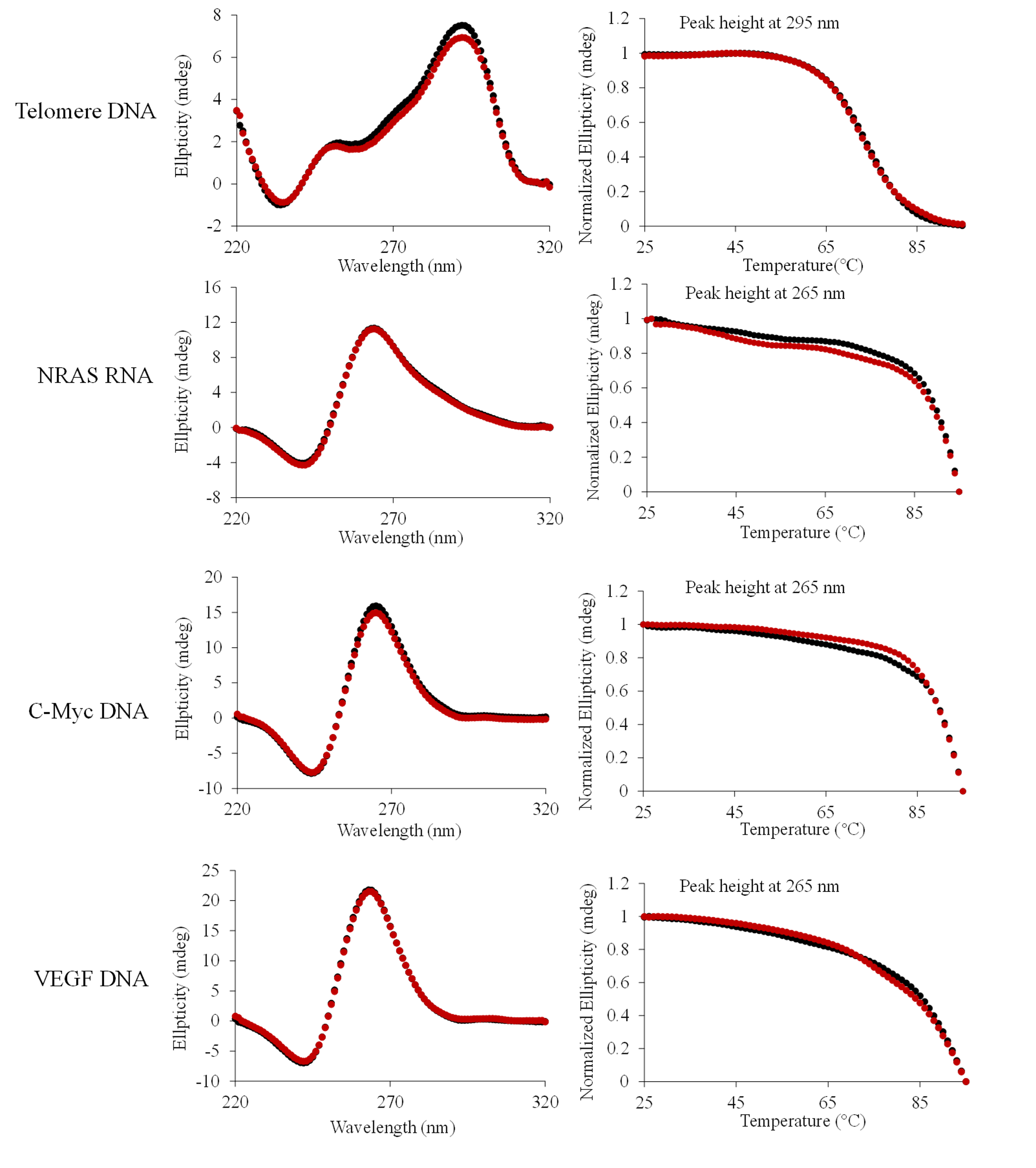
****Supplementary Figure 5.** Measurement of melting temperatures (Tm) by CD spectroscopy of DNA G-quadruplexes and RNA G-quadruplex, with (red circles) or without (black circles) OMT. Dataset of the ΔTm was showed downside, which was determined by subtracting the Tm value with OMT from the Tm value without OMT.

**Supplementary Figure 6.** Melting temperatures (Tm) of DNA double strand with (red circles) or without (black circles) OMT was measured by UV absorbance. The ΔTm was determined by subtracting the Tm value with OMT from the Tm value without OMT.


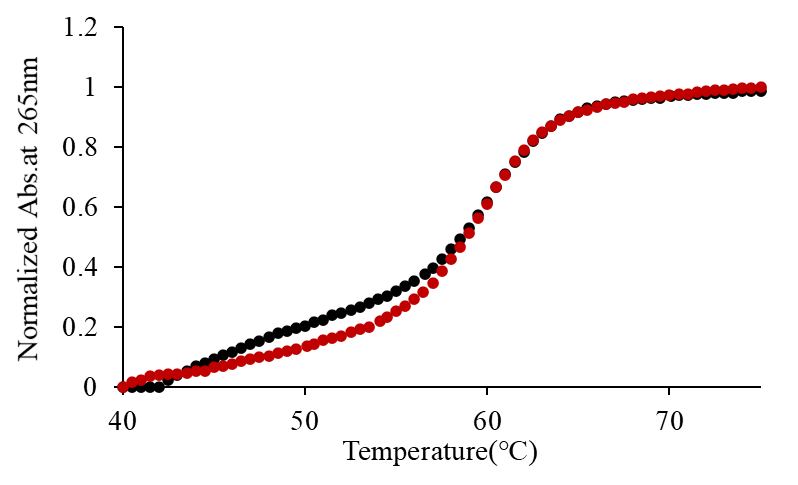

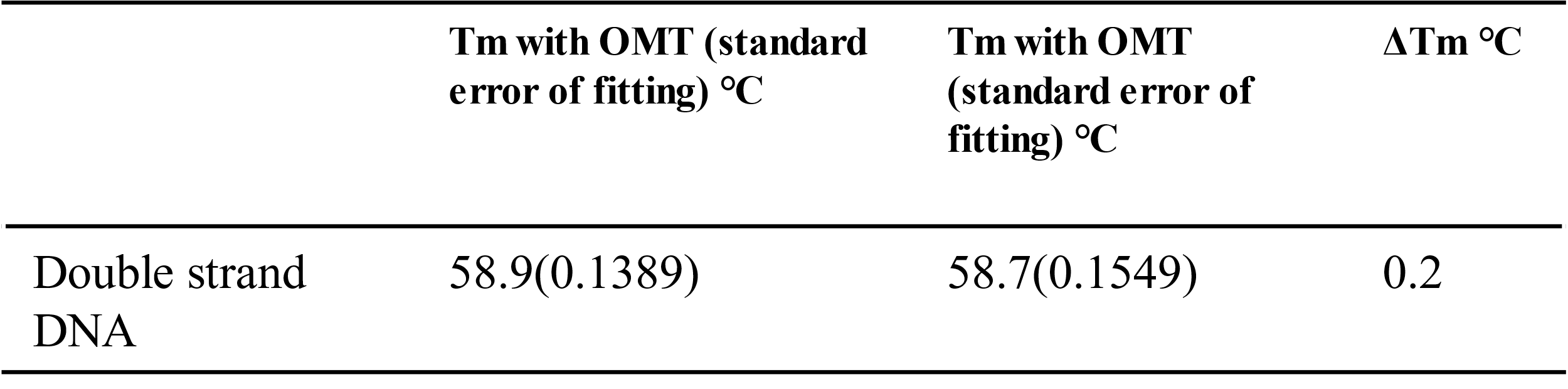


**
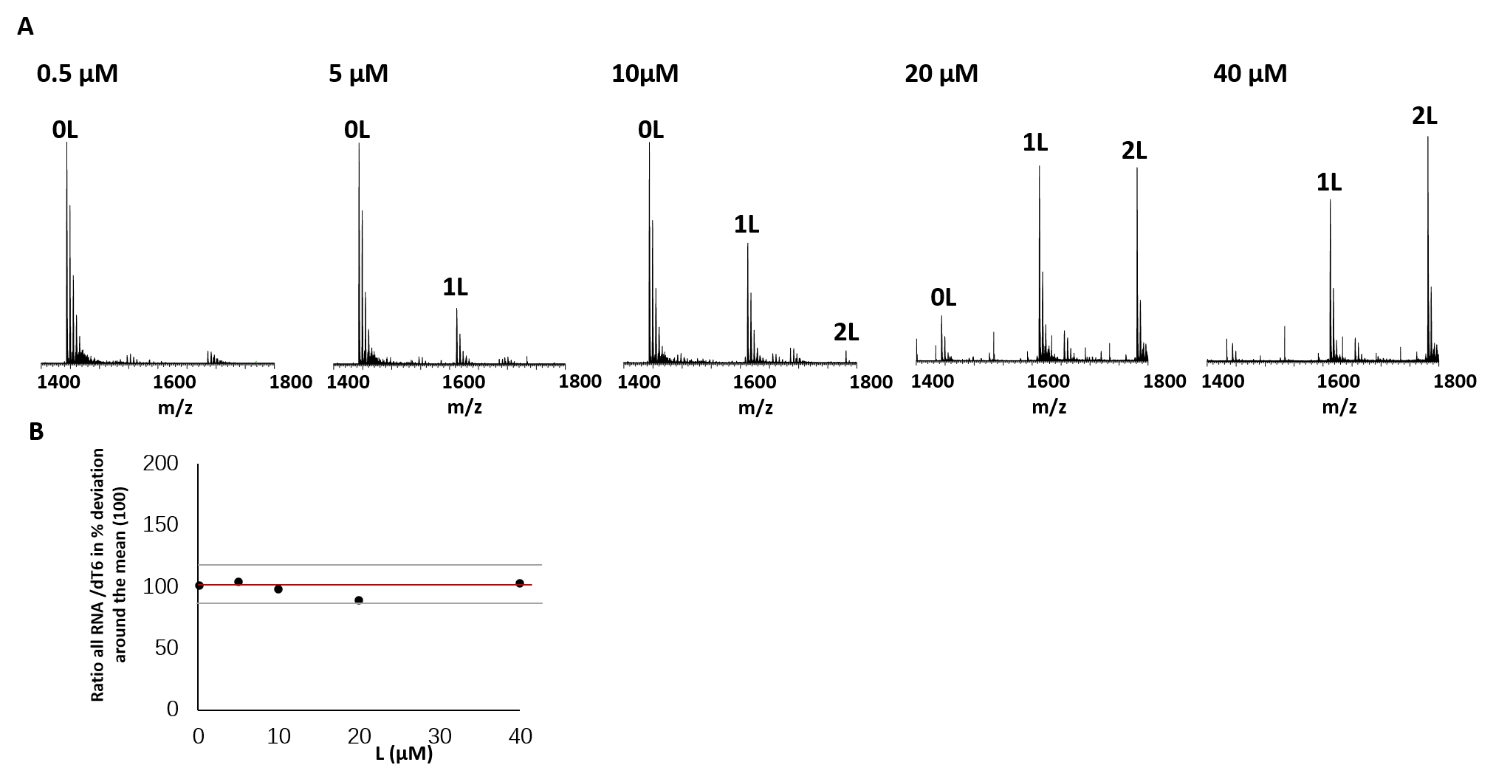
**

**Supplementary Figure 7.** TMPyp4 was used as the positive control for ESI-MS titration. **(A)** Titration of 10 µM VEGF RNA quadruplex with TMPyp4 at different concentrations. **(B)** Evolution of the ratio between the total RNA signal of the 4- charge state and the internal standard dT6 in function of the ligand concentration in percentage of deviation around the mean for VEGF RNA quadruplex with TMPyp4.

**
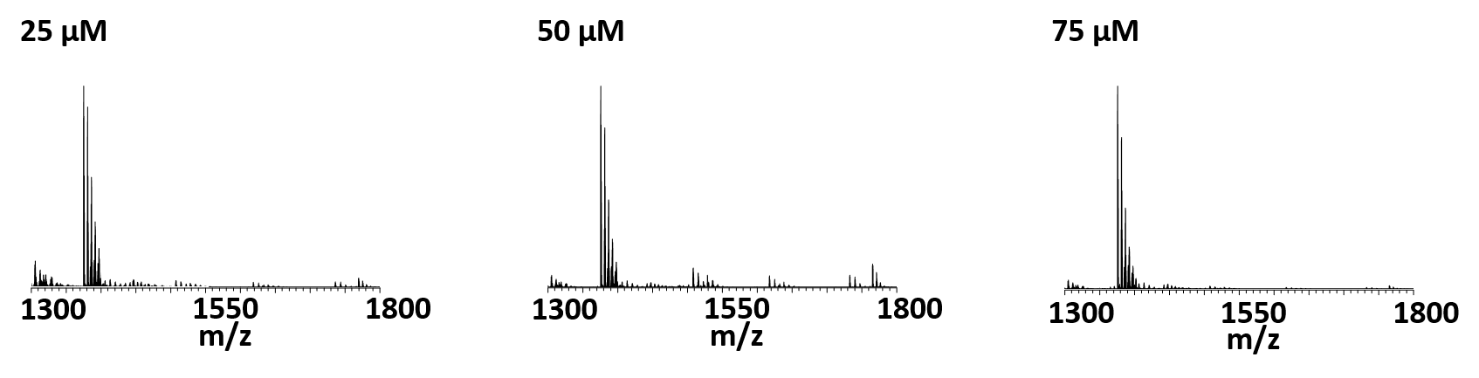
**

**Supplementary Figure 8.** ESI-MS spectra of mut 5 mixed with OMT at different concentrations.


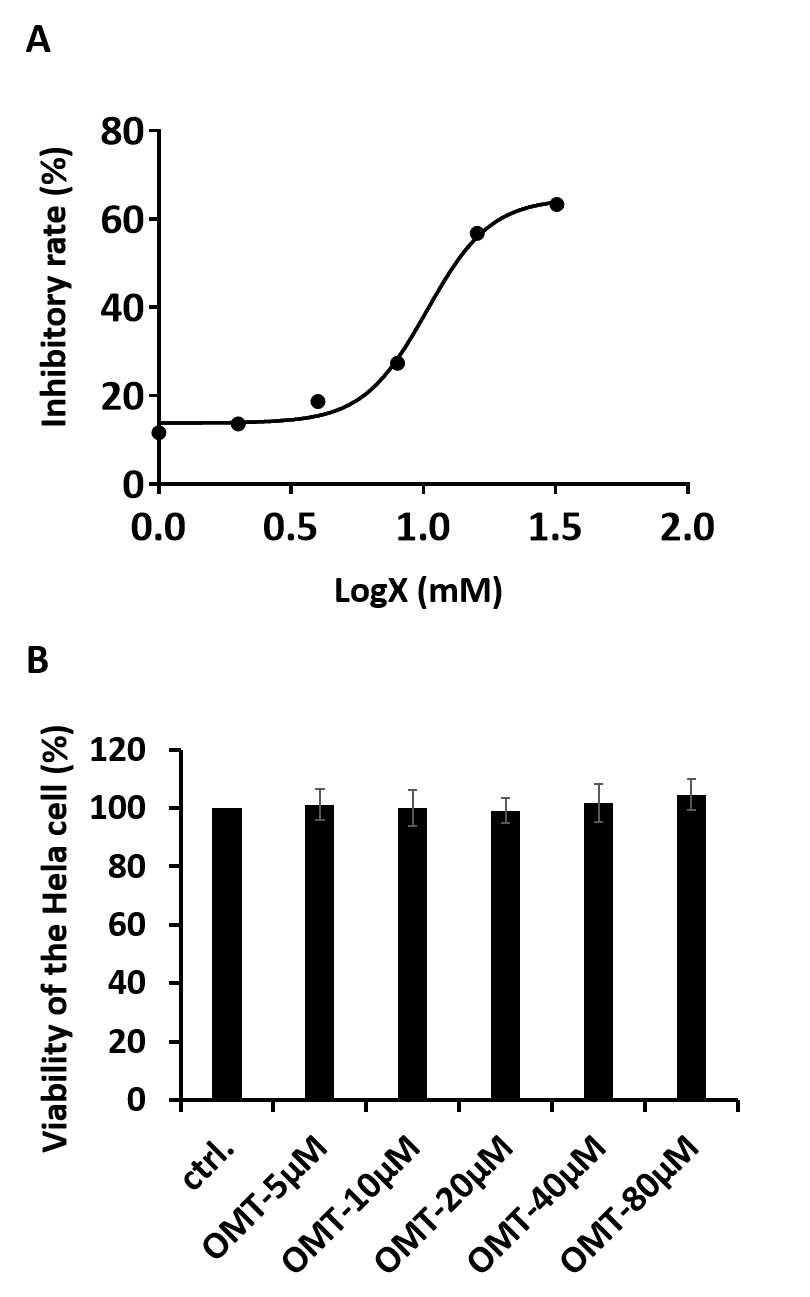
**Supplementary Figure 9.** Cell viability was ascertained by the standard CellTiter-Glo1 Luminescent Cell Viability Assay. **(A)** Dose response curve used to generate CC_50_ for OMT. **(B)** Percent viability of Hela cell treated with OMT at the concentration used in western blot analysis and luciferase assays.


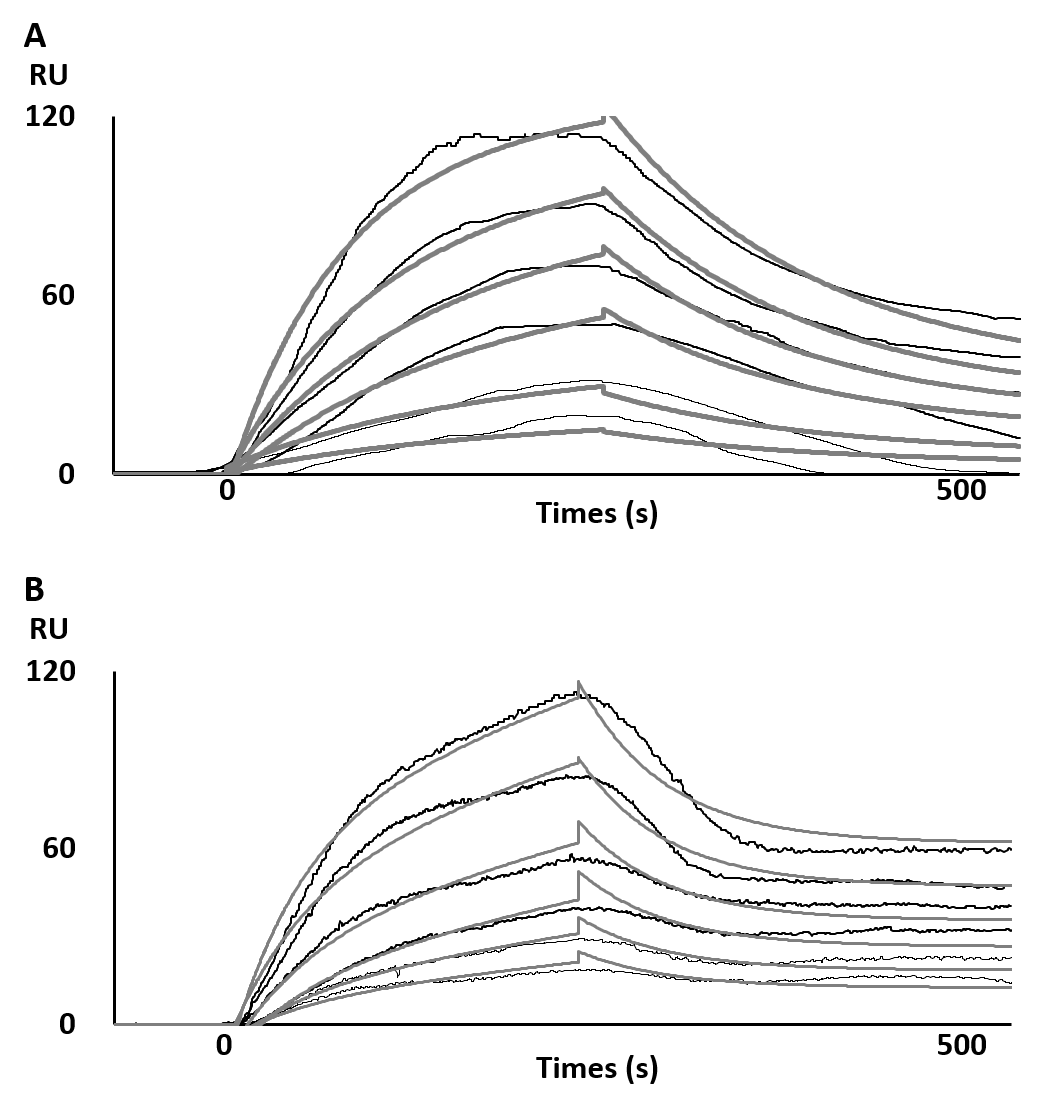
**Supplementary Figure 10.** SPR sensorgrams for binding of OMT with G-quadruplexes. Experimental data was in black and fitting curves were in gray. (A) The data of OMT binding with mut 1. The data was fitted with one-to-one model. (B) The data of OMT binding with mut 2.

***Table S1.*** RNA or DNA sequences used for in vitro experiments in this study.

| Templates in RTase reaction | Sequence (sequence forming G-quadruplex are underlined |
| --- | --- |
| VEGF | 5′-GCUAGCUCGGGCCGGGAGGAGCCGCAGCCAGAAGAGAAGGAAGAGGAGAGGGGGCCGCAGUGGCGACUCGGCGCUCGGAAGCCGGGCUCAUGGACGGGUGAGGCGGCGGUGUGCGCAGACGGAGGAGGGGGAGGAGGAAGUGCUCCAGCCGCGCGCGCUCCCCAGGCCCUGGCCCGGGCCUCGGGCCGGGGAGGAAGAGUAGCUCGCCGAGGCGCCGAGGAGAGCGGGCCGCCCCACAGCCCGAGCCGGAGAGGGAGCGCGAGCCGCGCCGGCCCCGGUCGGGCCUCCGAAACCAUG-3′ |
|  |  |
| BCL-2 | 5′-UAAUACGACUCACUAUAGGGCAUGCCAAGGGGGAAACACCAGAAUCAAGUGUUCCGCGUGAUUGAAGACACCCCCUCGUCCAAGAAUGCAAAGCACAUCCAGGGGGCCGUGGGGUGGGAGCUGGGGAUAAAAUAGCUGGAUUAUAACUCCUCUUCUUUCUCUCGAGAGGUGCCGUUGGCCCCCGUUGCUUUUCCUCUGGGAAGGAUG-3′ |

| For CD/UV experiments | sequence |
| --- | --- |
| DNA G-quadruplex(Telomere DNA) | 5′-AGGGTTAGGGTTAGGGTTAGGG-3′ |
| DNA G-quadruplex(c-myc) | 5′-TGAGGGTGGGTAGGGTGGGTAA-3′ |
| DNA G-quadruplex(VEGF) | 5′-CGGGGCGGGCCGGGGGCGGGGT-3′ |
| RNA G-quadruplex(NRAS) | 5′-UGUGGGAGGGGCGGGUCUGGG-3′ |
| RNA G-quadruplex(VEGF) | 5′-GGAGGAGGGGGAGGAGG-3′ |
| DNA double strand | 5′-GCGCGCGCGCGCGCGCGCGCGCGC-3′ |

| For SPR experiments | sequence |
| --- | --- |
| BCL-2 | 5′-biotin-GGGGGCCGUGGGGUGGGAGCUGGGG-3′ |
| NRAS | 5′-biotin-UGUGGGAGGGGCGGGUCUGGG-3′ |
| VEGF | 5′-biotin-GGAGGAGGGGGAGGAGG-3′ |
| mut 1 | 5′-biotin-GGAUGAGUGGGAGGAGG-3′ |
| mut 2 | 5′-biotin-UGAGGAGGGUGAGGAGG-3′ |
| mut 3 | 5′-biotin-GGAGGAGGGUGAGUAGG-3′ |
| mut 4 | 5′-biotin-UGAGGAGGGGGAGGAUG-3′ |
| mut 5 | 5′-biotin-UGAUGAGUGUGAGGAGG-3′ |

***Table S2.*** Sequences used for Plasmid Construction

| P1 | GCTTGGTACCGAGCTCGG |
| --- | --- |
| P2 | GTTTCCTTTGTTCTGGATCATAAACTTTCGAAGTCATGGTGGCGGATCCGAGCTCGGTACCAAGC |
| P3 | GTTTATGATCCAGAACAAAGGAAACGGATGATAACTGGTCCGCAGTGGTGGGCCAGATGTAAACA |
| P4 | TTTTTCTGAATCATAATAATTAATAAATGAATCAAGAACATTCATTTGTTTACATCTGGCCCACC |
| P5 | TGATTCATTTATTAATTATTATGATTCAGAAAAACATGCAGAAAATGCTGTTATTTTTTTACATG |
| P6 | CACAACATGTCGCCATAAATAAGAAGAGGCCGCGTTACCATGTAAAAAAATAACAGCATTTTCTG |
| P7 | CTTATTTATGGCGACATGTTGTGCCACATATTGAGCCAGTAGCGCGGTGTATTATACCAGACCTT |
| P8 | TATAAGAACCATTACCAGATTTGCCTGATTTGCCCATACCAATAAGGTCTGGTATAATACACCGC |
| P9 | GGCAAATCTGGTAATGGTTCTTATAGGTTACTTGATCATTACAAATATCTTACTGCATGGTTTGA |
| P10 | ATGGCCGACAAAAATGATCTTCTTTGGTAAATTAAGAAGTTCAAACCATGCAGTAAGATATTTGT |
| P11 | AAGATCATTTTTGTCGGCCATGATTGGGGTGCTTGTTTGGCATTTCATTATAGCTATGAGCATCA |
| P12 | ACTACACTTTCAGCGTGAACTATTGCTTTGATCTTATCTTGATGCTCATAGCTATAATGAAATGC |
| P13 | AGTTCACGCTGAAAGTGTAGTAGATGTGATTGAATCATGGGATGAATGGCCTGATATTGAAGAAG |
| P14 | AACCATTTTTTCTCCTTCTTCAGATTTGATCAACGCAATATCTTCTTCAATATCAGGCCATTCAT |
| P15 | ATCTGAAGAAGGAGAAAAAATGGTTTTGGAGAATAACTTCTTCGTGGAAACCATGTTGCCATCAA |
| P16 | CAAGATATGCTGCAAATTCTTCTGGTTCTAACTTTCTCATGATTTTTGATGGCAACATGGTTTCC |
| P17 | CAGAAGAATTTGCAGCATATCTTGAACCATTCAAAGAGAAAGGTGAAGTTCGTCGTCCAACATTA |
| P18 | CAGGTTTACCACCTTTTACTAACGGGATTTCACGAGGCCATGATAATGTTGGACGACGAACTTCA |
| P19 | CGTTAGTAAAAGGTGGTAAACCTGACGTTGTACAAATTGTTAGGAATTATAATGCTTATCTACGT |
| P20 | CCGATTCAATAAACATTTTTGGTAAATCATCACTTGCACGTAGATAAGCATTATAATTCCTAACA |
| P21 | ATTTACCAAAAATGTTTATTGAATCGGACCCAGGATTCTTTTCCAATGCTATTGTTGAAGGTGCC |
| P22 | TGAAGACCTTTTACTTTGACAAATTCAGTATTAGGAAACTTCTTGGCACCTTCAACAATAGCATT |
| P23 | GAATTTGTCAAAGTAAAAGGTCTTCATTTTTCGCAAGAAGATGCACCTGATGAAATGGGAAAATA |
| P24 | TATTGTTCATTTTTGAGAACTCGCTCAACGAACGATTTGATATATTTTCCCATTTCATCAGGTGC |
| P25 | AGCGAGTTCTCAAAAATGAACAATAAGCTAGCTCGGGCCGGGAGGAGCCGCAGCCGGAGGAGGGG |
| P26 | CCGAGTCGCCACTGCGGCCCCCTCTCCTCTTCCTTCTCTTCTTCCTCCTCCCCCTCCTCCGGCTG |
| P27 | CGCAGTGGCGACTCGGCGCTCGGAAGCCGGGCTCATGGACGGGTGAGGCGGCGGTGTGCGCAGAC |
| P28 | CGGCCCGAGGCCCGGGCCAGGGCCTGGGGAGCGCGCGCGGCTGGAGCACTGTCTGCGCACACCGC |
| P29 | CGGGCCTCGGGCCGGGGAGGAAGAGTAGCTCGCCGAGGCGCCGAGGAGAGCGGGCCGCCCCACAG |
| P30 | TCGGAGGCCCGACCGGGGCCGGCGCGGCTCGCGCTCCCTCTCCGGCTCGGGCTGTGGGGCGGCCC |
| P31 | CGGTCGGGCCTCCGAAACCATGATGGAAGACGCCAAAAACATAAAGAAAGGCCCGGCGCCATTCT |
| P32 | CTTCATAGCCTTATGCAGTTGCTCTCCAGCGGTTCCATCTTCCAGCGGATAGAATGGCGCCGGGC |
| P33 | GCAACTGCATAAGGCTATGAAGAGATACGCCCTGGTTCCTGGAACAATTGCTTTTACAGATGCAC |
| P34 | ACATTTCGAAGTACTCAGCGTAAGTGATGTCCACCTCGATATGTGCATCTGTAAAAGCAATTGTT |
| P35 | ACGCTGAGTACTTCGAAATGTCCGTTCGGTTGGCAGAAGCTATGAAACGATATGGGCTGAATACA |
| P36 | GAATTGAAGAGAGTTTTCACTGCATACGACGATTCTGTGATTTGTATTCAGCCCATATCGTTTCA |
| P37 | TGCAGTGAAAACTCTCTTCAATTCTTTATGCCGGTGTTGGGCGCGTTATTTATCGGAGTTGCAGT |
| P38 | GTTGAGCAATTCACGTTCATTATAAATGTCGTTCGCGGGCGCAACTGCAACTCCGATAAATAACG |
| P39 | TTATAATGAACGTGAATTGCTCAACAGTATGGGCATTTCGCAGCCTACCGTGGTGTTCGTTTCCA |
| P40 | GATTGGGAGCTTTTTTTGCACGTTCAAAATTTTTTGCAACCCCTTTTTGGAAACGAACACCACGG |
| P41 | GTGCAAAAAAAGCTCCCAATCATCCAAAAAATTATTATCATGGATTCTAAAACGGATTACCAGGG |
| P42 | GGAGGTAGATGAGATGTGACGAACGTGTACATCGACTGAAATCCCTGGTAATCCGTTTTAGAATC |
| P43 | CGTCACATCTCATCTACCTCCCGGTTTTAATGAATACGATTTTGTGCCAGAGTCCTTCGATAGGG |
| P44 | GACCAGTAGATCCAGAGGAGTTCATGATCAGTGCAATTGTCTTGTCCCTATCGAAGGACTCTGGC |
| P45 | ACTCCTCTGGATCTACTGGTCTGCCTAAAGGTGTCGCTCTGCCTCATAGAACTGCCTGCGTGAGA |
| P46 | TCCGGAATGATTTGATTGCCAAAAATAGGATCTCTGGCATGCGAGAATCTCACGCAGGCAGTTCT |
| P47 | TGGCAATCAAATCATTCCGGATACTGCGATTTTAAGTGTTGTTCCATTCCATCACGGTTTTGGAA |
| P48 | GACGACTCGAAATCCACATATCAAATATCCGAGTGTAGTAAACATTCCAAAACCGTGATGGAATG |
| P49 | GATATGTGGATTTCGAGTCGTCTTAATGTATAGATTTGAAGAAGAGCTGTTTCTGAGGAGCCTTC |
| P50 | AGAATAGGGTTGGCACCAGCAGCGCACTTTGAATCTTGTAATCCTGAAGGCTCCTCAGAAACAGC |
| P51 | CTGGTGCCAACCCTATTCTCCTTCTTCGCCAAAAGCACTCTGATTGACAAATACGATTTATCTAA |
| P52 | AGAGAGGGGAGCGCCACCAGAAGCAATTTCGTGTAAATTAGATAAATCGTATTTGTCAATCAGAG |
| P53 | TGGCGCTCCCCTCTCTAAGGAAGTCGGGGAAGCGGTTGCCAAGAGGTTCCATCTGCCAGGTATCA |
| P54 | TAATCAGAATAGCTGATGTAGTCTCAGTGAGCCCATATCCTTGCCTGATACCTGGCAGATGGAAC |
| P55 | TGAGACTACATCAGCTATTCTGATTACACCCGAGGGGGATGATAAACCGGGCGCGGTCGGTAAAG |
| P56 | GTTTTCCCGGTATCCAGATCCACAACCTTCGCTTCAAAAAATGGAACAACTTTACCGACCGCGCC |
| P57 | GGATCTGGATACCGGGAAAACGCTGGGCGTTAATCAAAGAGGCGAACTGTGTGTGAGAGGTCCTA |
| P58 | GGCGTTGGTCGCTTCCGGATTGTTTACATAACCGGACATAATCATAGGACCTCTCACACACAGTT |
| P59 | GGAAGCGACCAACGCCTTGATTGACAAGGATGGATGGCTACATTCTGGAGACATAGCTTACTGGG |
| P60 | CAGAGACTTCAGGCGGTCAACGATGAAGAAGTGTTCGTCTTCGTCCCAGTAAGCTATGTCTCCAG |
| P61 | TGACCGCCTGAAGTCTCTGATTAAGTACAAAGGCTATCAGGTGGCTCCCGCTGAATTGGAATCCA |
| P62 | AGACCTGCGACACCTGCGTCGAAGATGTTGGGGTGTTGGAGCAAGATGGATTCCAATTCAGCGGG |
| P63 | GCAGGTGTCGCAGGTCTTCCCGACGATGACGCCGGTGAACTTCCCGCCGCCGTTGTTGTTTTGGA |
| P64 | GGCGACGTAATCCACGATCTCTTTTTCCGTCATCGTCTTTCCGTGCTCCAAAACAACAACGGCGG |
| P65 | GATCGTGGATTACGTCGCCAGTCAAGTAACAACCGCGAAAAAGTTGCGCGGAGGAGTTGTGTTTG |
| P66 | TTTCTTGCGTCGAGTTTTCCGGTAAGACCTTTCGGTACTTCGTCCACAAACACAACTCCTCCGCG |
| P67 | GGAAAACTCGACGCAAGAAAAATCAGAGAGATCCTCATAAAGGCCAAGAAGGGCGGAAAGATCGC |
| P68 | GTGCTGGATATCTGCAGAATTCTTACACGGCGATCTTTCCGCCCTTC |

***Table S3.***

|  | *K_a_*(M^-1^S^-1^) | *K_d_*(S^-1^) | *K_D_*(M) |  |
| --- | --- | --- | --- | --- |
| VEGF | 9.76×10^-1^ | 3.66×10^-3^ | 3.75×10^-5^ |  |
|  |  |  |  |  |
| mut 1 | 6.76×10^-1^ | 2.64×10^-3^ | 3.90×10^-5^ |  |
| mut 2 | 4.90×10^-1^ | 4.95×10^-3^ | 1.01×10^-4^ |  |

*K_d_*: The dissociation rate constant; *K_a_*: The association rate constant.
